# The Epithelial Barrier Hypothesis in Food Allergies: The State of the Art

**DOI:** 10.3390/nu17061014

**Published:** 2025-03-14

**Authors:** Valentina Agnese Ferraro, Stefania Zanconato, Silvia Carraro

**Affiliations:** Unit of Pediatric Allergy and Respiratory Medicine, Women’s and Children’s Health Department, University of Padova, 35128 Padova, Italy; valentinaagnese.ferraro@unipd.it (V.A.F.); stefania.zanconato@aopd.veneto.it (S.Z.)

**Keywords:** food allergy, epithelial barrier hypothesis, epithelial barrier damage

## Abstract

Recently, the “epithelial barrier hypothesis” has been proposed as a key factor in the development of allergic diseases, such as food allergies. Harmful environmental factors can damage epithelial barriers, with detrimental effects on the host immune response and on the local microbial equilibrium, resulting in chronic mucosal inflammation that perpetuates the dysfunction of the epithelial barrier. The increased epithelial permeability allows allergens to access the submucosae, leading to an imbalance between type 1 T-helper (Th1) and type 2 T-helper (Th2) inflammation, with a predominant Th2 response that is the key factor in food allergy development. In this article on the state of the art, we review scientific evidence on the “epithelial barrier hypothesis”, with a focus on food allergies. We describe how loss of integrity of the skin and intestinal epithelial barrier and modifications in gut microbiota composition can contribute to local inflammatory changes and immunological unbalance that can lead to the development of food allergies.

## 1. Introduction

The prevalence of allergic diseases has been rising since the 1960s. Recently, the “epithelial barrier hypothesis” has been emphasized as a key factor in the development of these conditions [1]. First described in 2017 by Pothoven and Schleimer [2], this hypothesis states that epithelial barrier damage due to increased exposure to agents associated with industrialization, urbanization, and contemporary living is responsible for the dramatically increased prevalence of allergic disorders in recent decades [1,3]. Every day, new potentially hazardous chemicals enter our lives, adversely affecting the epithelial tissues, microbiome, immune system, and human health [4]. 

The evidence indicates that epithelial barrier dysfunction can be seen both as a driver of the abnormal immune response to environmental triggers and as a result of persistent inflammation induced by exposure to hazardous environmental agents [5]. In fact, damage to the epithelial barrier caused by environmental toxins leads to an imbalance in the microbiota, triggers chronic inflammation with the activation of immune responses, and impairs the healing of the epithelial barrier [1].

Among allergic disorders in which dysfunctional epithelial barriers are involved, the literature has described eosinophilic oesophagitis, atopic dermatitis, allergic rhinitis, allergic asthma, and food allergies [6,7,8].

It has been hypothesized that a damaged epithelial barrier because of environmental factors, such as infections, changes in diet, increased pollution, and intensive hygiene, plays a crucial role in the development of food allergies [9,10,11]. In turn, a damaged epithelial barrier induces microbial dysbiosis, characterized by the colonization of opportunistic pathogens and the loss of commensals, followed by abnormal interactions with the host immune system, resulting in chronic mucosal inflammation [12].

In this article on the state of the art, we review scientific evidence on the “epithelial barrier hypothesis”, with a particular emphasis on its impact on the development of food allergies. We explore how disruptions in the epithelial barrier, influenced by environmental factors and host immune interactions, can contribute to allergic sensitization and the onset of food allergies.

## 2. Materials and Methods

The MEDLINE/Pubmed database was searched for this narrative review, using the following terms: “epithelial barrier hypothesis” or “epithelial barrier” and “food allergy” or “food allergies”.

Firstly, we analyzed the role of the epithelial barrier, the factors that contribute to epithelial damage, and the mechanisms underlying this injury. Then, we focused on the role of epithelial barrier dysfunction in food allergies.

The filters applied were language (English) and age of study subjects (0–18 years).

## 3. Results

### 3.1. The Role of the Epithelial Barrier

The internal and external surfaces of the human body are covered by epithelial tissue, which is essential to protect the structural and functional integrity of organisms, acting as the first defense line against any potentially harmful elements from the outside [13]. The epithelium acts as selective barrier, as it facilitates nutrient, gas, and water exchange while concurrently blocking the infiltration of microbes, toxins, allergens, and chemical agents from the environment [14]. Moreover, the epithelium regulates microbiome homeostasis and contributes to the development and maintenance of immune tolerance [15].

To carry out these tasks, epithelial cells are connected through intercellular junctions, which are responsible for maintaining the integrity of the mucosal barrier. Tight junctions (TJs), adherens junctions (AJs), and desmosomes work together to ensure stable intercellular adhesion, preventing the movement of soluble substances, proteins, and pathogens between the apical and basolateral surfaces [16,17]. Moreover, the mucus layer, located on top of epithelial cells, plays as an effective barrier against harmful substances being rich in antimicrobial peptides and immunoglobulin A [18].

Despite having the same function, the epithelial barriers of the skin and the gastrointestinal and respiratory tracts exhibit differences in terms of structure, function, and biochemical properties.

The epidermis provides a robust physical barrier largely reliant on the stratum corneum, which is the outermost layer formed through the differentiation of keratinocytes. These cells are essential to maintain the skin’s protective barrier, mainly through the production of filaggrin (FLG), loricrin, and keratin filaments [14]. Other types of epidermal cells include Langerhans cells, which are part of the tissue-resident macrophage family and play a role in regulating skin homeostasis and immune responses to environmental stimuli [19].

The intestinal epithelium consists of columnar cells, mostly enterocytes, which are absorptive cells with microvilli, and goblet cells, which produce mucus to protect the epithelium from substances in the intestinal lumen. Moreover, the intestinal epithelium has Paneth cells (producing antimicrobial peptides), tuft cells (chemosensory cells involved in the immune response), and stem cells (proliferating continuously to replace the epithelium) [20].

The airway epithelium changes from a pseudostratified structure in the upper airway to a single layer of squamous epithelial cells in the alveoli [21]. The airway epithelium includes goblet cells and basal stem cells, and it is covered with cilia, whose coordinated movements clear the epithelium and prevent the accumulation of foreign materials or mucus on the surface of epithelial cells [22].

Apart from their role as a physical barrier, epithelial cells are actively involved in chemical defense through the secretion of antimicrobial peptides, proteases, and antioxidants, which contribute to pathogen neutralization [23]. Epithelial cells, indeed, have molecular sensors that, after detecting specific ligands on a microbic surface, initiate signaling pathways that result in the production and release of pro-inflammatory cytokines and chemokines. These signaling molecules play a pivotal role in the immune response by attracting and activating various cells of the innate and adaptive immune systems, thereby orchestrating a coordinated defense against infections [24,25].

In summary, the epithelium plays a crucial role in maintaining the functional and structural integrity of the tissues, thereby contributing to the overall health of the organism.

### 3.2. Factors That Cause Epithelial Barrier Damage

In the last decades, a large number of new chemical substances have been introduced into human environments, with minimal regulation regarding their health impact [26]. This shift in environmental exposure has been hypothesized to be closely associated with the increasing prevalence of allergic diseases [27]. Among the factors contributing to environmental damage with a significant impact on human health, it is essential to include the following: climate change, ozone, air pollutants such as particulate matter, tobacco smoke, detergents, household cleaners, microplastics (MPs) and nanoplastics (NPs), dietary fatty acids, and processed food [28]. All these factors can damage the integrity of epithelial barriers, which are crucial for maintaining the body’s defense mechanisms against environmental insults [29].

Climate change presents a critical challenge on a global scale. It is largely driven by human activities, which reduces the Earth’s natural ability to absorb and eliminate carbon dioxide (CO2), resulting in severe consequences for human health [30,31]. Moreover, climate change leads to more frequent wildfires, whose smoke contains toxic substances, and higher temperatures, which accelerate ozone production, causing oxidative stress and damaging the epithelial barrier [32,33]. Acute ozone exposure impairs airway epithelial barrier function, inducing inflammation, airway hyperreactivity, and the release of IL (interleukin)-1a and IL-33 [34,35].

Also, air pollutants significantly contribute to epithelial barrier impairment, mainly activating the secretion of alarmins through the damage of epithelial cells [33]. Particulate matter (PM), specifically, is a mixture of solid particles and liquid droplets. Such inhalable particles can go deep into the lungs, where they cause respiratory diseases [36], mainly altering epithelial barrier structure by degrading TJs [37].

Exposure to cigarette smoke is a further cause of increased permeability of the epithelial barrier because it reduces the expression of TJ proteins and leads to the breakdown of intercellular junctions [38]. Moreover, cigarette smoke induces the activation of dendritic cells in the lung, which upregulate inflammation against an effective immune response [39]. In recent years, e-cigarettes have also been associated with epithelial damage. Indeed, e-cigarettes release vaporized nicotine and flavorings, both of which contain multiple toxic substances known to damage the epithelial barrier [40].

Detergents, particularly those containing surfactants like sodium lauryl sulfate and sodium benzene sulphonate, have been widely used in our daily lives since the 1960s, but only recently they have been identified as significant disruptors of the epithelial barrier [41]. Even at very high dilutions, laundry detergents have been shown to compromise epithelial barrier function in both skin and bronchial epithelial cells, leading to increased vulnerability to external irritants and allergens [42,43]. Furthermore, since the year 2000, professional dishwashing has become standard in common food consumption areas, such as hospitals and schools. The rinse aids used by these machines often contain alcohol ethoxylates that have been found to open the epithelial barrier and exhibit pro-inflammatory and cytotoxic effects on gut epithelial cells, even at very low concentrations [44].

MPs and NPs are small fragments and particles from plastic waste that infiltrate food and water supplies, posing significant health risks [45]. The small size of MPs and NPs enables them to penetrate tissues and to interact with cells and cellular structural components [46]. Molecular simulations have shown that NPs dissolve in epithelial cells within the hydrophobic core of the lipid bilayer, leading to structural and dynamic alterations that affect the cell membrane [47]. Moreover, they have been shown to induce epithelial inflammation, especially in the gut, compromising the integrity of the epithelial barrier [45].

As far as the intestinal epithelium is concerned, a threat to the epithelial barrier is represented by exposure to high intake of dietary fatty acids and processed foods and poor intake of antioxidant-containing foods. In processed foods, the use of food additives such as synthetic colorants, emulsifiers (polysorbates 20 and 80), and advanced glycation end-products formed during heat processing, can disrupt the integrity of the epithelial barrier, altering the microbiome and activating the immune system [48,49]. The literature shows that food emulsifiers induce cellular toxicity, induce transcriptome alterations, and changed protein expression in gastrointestinal epithelial cells [50]. Recently, a European Academy of Allergy & Clinical Immunology (EAACI) task force report [51] provided evidence on the potential role of ultra-processed foods in facilitating the development of allergic disorders, primarily due to higher exposure to fructose, carbonated soft drinks, and free sugar intake. Experts highlighted the association between the intake of commercial baby food during infancy, such as fruit juices, sugar-sweetened beverages, high-carbohydrate ultra-processed foods, monosodium glutamate, and advanced glycated end-products, and confirmed childhood food allergies [51].

In summary, the continuous exposure to all these environmental substances poses significant threats to the integrity of the epithelial barrier. This disruption is mostly linked to the opening of the tight-junction barriers that induce inflammation, cell death, and oxidative stress.

### 3.3. Mechanisms of Epithelial Barrier Damage

**Figure 1:**
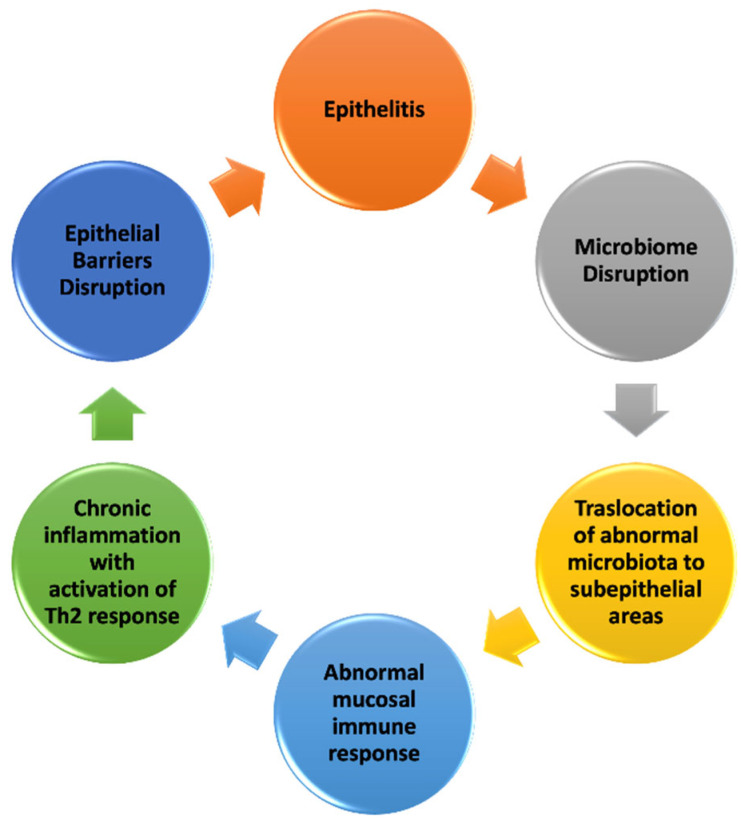
Mechanisms of epithelial barrier damage (modified from [15,29,52]).

The complex interplay between the harmful factors described above and the epithelial barrier results in the loss of normal structure integrity and barrier function. Understanding the mechanisms underlying epithelial barrier dysfunction is crucial (Figure 1).

Barrier integrity is firstly maintained by mucus and antimicrobial peptides localized on the surfaces of the epithelium [52]. In addition, a relevant role is played by TJs, located on the apical–lateral surface of epithelial cells. TJs consist of multiprotein complexes formed by transmembrane proteins, such as claudins, occludins, and junctional adhesion molecules (JAMs) and adaptor proteins (zonula occludens and cingulin) [16,53]. Claudins, which reside in the transmembrane area, are the major controllers of selective permeability [16].

Once the epithelial barrier is damaged by environmental noxae, its integrity is lost, leading to the onset of epithelitis, characterized by epithelial inflammation and epithelial damage. Agents such as PM, ozone, detergents, and dishwasher rinse aids cause epithelial cell damage and stimulate the release of thymic stromal lymphopoietin (TSLP), IL-25, and IL-33 from the epithelial cells [4]. The literature reports a disruption in the microbiome, with the colonization of opportunistic pathogens followed by the translocation of microbiota to subepithelial areas [54]. Aberrant host microbiota and epithelial barrier interactions lead to abnormal mucosal immune responses, including the upregulation of T-helper 17 (Th17) cells and the downregulation of T regulatory cells [55]. This is followed by immune system activation, inducing cell death, cellular stress, altered expression of cell adhesion molecules, and increased inflammation. The immune system response is characterized by cytokine production, leading to the activation of Th2 inflammation with the production of type 2 cytokines such as IL-4, IL-5, IL-13, and GM-CSF (granulocyte–macrophage colony-stimulating factor), B cells undergoing isotype switch to IgE, and the activation of eosinophils [4]. In a recent study, authors showed that the intranasal administration of two commercial laundry detergents and two commonly used surfactants for cleaning and cosmetics (SLS and sodium dodecyl benzene sulfonate) in mice induced eosinophilic airway inflammation with increased IL-33 expression and the activation of group 2 innate lymphoid cells (ILC2s) [56].

Once the type 2 immune response has been activated, the epithelium cannot fully repair and close the barrier, leading to a vicious cycle of leaky barriers, microbial dysbiosis, and chronic inflammation [57] (Figure 1). This shift in the immune response towards a type 2 profile provides the foundation for the development of allergic diseases, occurring in a favorable environment for allergen sensitization due to persistent epithelial barrier dysfunction and chronic inflammation [58].

### 3.4. Epithelial Barrier Dysfunction and Food Allergies

Food allergies, which are associated with reduced quality of life and increased healthcare costs, affect approximately 5–8% of the global population, and their prevalence is rising each year [59]. Recently, food allergies have been recognized as a disease potentially linked to epithelial barrier dysfunction [9]. In animal models of food allergies, mice are sensitized to ovalbumin or other allergens by epicutaneous administration of the allergens [60]. While the defective epithelial barrier theory is robustly supported for the skin, its applicability to the gut epithelium remains less conclusive [61]. Evidence suggests that tolerance to food allergens typically develops through exposure in the gastrointestinal tract [62] and that a compromised intestinal epithelial barrier can result in sensitization to food allergens [63,64]. Moreover, together with impaired epithelial barrier function, food allergies result from alterations in the gut microbiome [65].

#### 3.4.1. Mechanisms of Epithelial Barrier Dysfunction in Food Allergies

The skin is the first barrier involved in food allergy pathogenesis [66]. It is well known that skin integrity, often compromised in diseases like atopic dermatitis, plays a fundamental role in food sensitization and food allergy development [10]. Lower levels of TJ protein are expressed in the skin of patients with food allergies [67]. Also, genetic mutations affecting skin barrier proteins, such as FLG and serine protease inhibitor Kazal type 5 (SPINK5), can significantly impair the skin’s protective function, leading to food allergies [68]. Loss-of-function mutations in FLG, a protein crucial for maintaining epidermal integrity, are linked to atopic dermatitis and increased food allergen sensitization [69]. Similarly, polymorphisms in the SPINK5 gene, which lead to abnormal skin keratinization and elevated levels of cytokines such as TSLP, are reported to be associated with food allergies [70]. Taken together, these data strongly support the role of barrier dysfunction in food allergy development.

**Figure 2:**
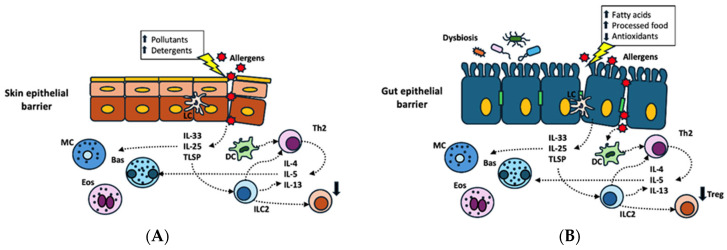
Skin (**A**) and gut (**B**) epithelial barrier dysfunction induced by different environmental toxic substances ((**A**) pollutants, detergents; (**B**) high intake of dietary fatty acids and processed foods and poor intake of antioxidant-containing foods) and disruption in gut microbiota (**B**). Allergens entering from damaged epithelium are captured by Langerhans cells (LCs) and dermal dendritic cells (DCs), with activation of T-helper 2 cells (Th2). Damaged epithelial cells release interleukin-25 (IL-25), interleukin-33 (IL-33), and thymic stromal lymphopoietin (TSLP), which activate innate lymphoid cells (ILC2s), eosinophils (EOS), basophils (Bas), and mast cells (MCs). ILC2s and Th2 cells secrete interleukin-4 (IL-4), interleukin-5 (IL-5), and interleukin-13 (IL-13), which induce an inflammatory immune response that plays a key role in allergic reactions.

The compromised skin barrier allows allergens to penetrate more easily (see Figure 2A). Allergens entering from damaged skin are captured by Langerhans cells (LCs) and dermal dendritic cells (DCs), which migrate to the nearby lymph nodes (LNs) to present the allergens to T cells. While LCs have a role in promoting tolerance and type 3 immune responses mediated by Th17 cells, dermal DCs stimulate T-helper 2 (Th2) cells, which are crucial in allergic responses [71]. Damaged epithelial cells release IL-25, IL-33, and TSLP, which lead to the activation of ILC2s, eosinophils (EOS), basophils (Bas), and mast cells (MCs). ILC2s and Th2 cells secrete IL-4, IL-5, and IL-13, which induce an inflammatory immune response that plays a key role in allergic reactions [58].

The intestinal epithelium is naturally structured to play a protective role against allergies by absorbing nutrients and facilitating contact with the immune system through a process that induces immune tolerance [72]. Such function is compromised in food allergies (see Figure 2B). It has been shown that 96% of patients with food allergies exhibit both structural and functional intestinal barrier defects, which were observed in the terminal ileum and two colorectal sites through confocal laser endomicroscopy [73]. As for structural defects, the intestinal epithelium of patients with food allergies expresses lower levels or damaged TJ protein [67]. The resulting increased intestinal permeability allows allergens to access the submucosae, leading to an imbalance between Th1 and Th2 inflammation with a predominant Th2 response in the presence of the interleukins IL-4, IL-5, and IL-13 [74]. Damaged epithelial cells release inflammatory cytokines, such as TSLP, IL-25, and IL-33, inducing the shift of the immune response from tolerance to hypersensitivity [75].

From an etiological standpoint, there is evidence suggesting that environmental factors, such as dietary fatty acids and processed food, can be responsible for intestinal mucosal barrier damage [76].

#### 3.4.2. Disruptions in Gut Microbiota and Food Allergies

Recent evidence suggests the role of gut microbiota in preserving the integrity of the intestinal epithelial barrier. Alterations in its composition may strongly affect the correct function of epithelial barriers, favoring the development of allergic diseases [77] (Figure 2B). Both in vitro and in vivo studies have highlighted a connection between the use of emulsifiers and the disruption of intestinal microbial homeostasis, which, in turn, stimulates innate immunity, promotes the degradation and thickening of the mucus layer, and increases intestinal permeability [51].

The role of the gut microbiome in the pathogenesis of food allergies has been demonstrated in microbial transfer experiments in mice, where a specific gut microbiota signature transmitted susceptibility to food allergies [78]. Moreover, several studies show that children with food allergies have an altered gut microbial composition compared to children without this condition [79,80].

This has been recently confirmed by a study showing that the gut microbiome signature in allergic children is different from that of healthy controls [81]. In addition, the authors demonstrated that the gut metagenome of allergic children exhibited a pro-inflammatory profile, characterized by an enrichment of genes involved in the production of bacterial lipopolysaccharides and urease [81]. These mechanisms significantly influence immune tolerance mechanisms and trigger type 2 inflammation, which is the main driver of food allergies [81,82].

The gut microbiota also has a role in the regulation of enteric eosinophils function, which, in turn, affects tissue repair and induces allergic sensitization to food antigens [83]. Jimenez-Saiz et al. [83] demonstrated that germ-free mice exhibit significantly increased eosinophilia in the small intestine compared to controls. Furthermore, they found that eosinophil-deficient mice developed intestinal fibrosis and showed a reduced tendency for allergic sensitization, highlighting the role of eosinophils in gut immune regulation [83].

#### 3.4.3. Airway Epithelial Barrier Dysfunction and Food Allergies

Also, airway epithelial barrier dysfunction may have a role in the pathogenesis of food allergies. Despite several case reports suggesting the potential sensitization to food antigens through the airways [84,85], how the interaction between food allergens and airway epithelial cells might influence the sensitization process is not yet understood. Recently, Palladino et al. [86] demonstrated that peanut allergens can disrupt the integrity of the airway epithelium and induced inflammation. The authors also highlighted the significant role of peanut lipids in regulating the number of allergens that can cross the epithelial barrier. Even if the direct influence of allergens on epithelial cells is not fully understood, this emerging evidence suggests that allergens per se can have a role in disrupting barrier integrity and triggering the immune response. Further studies are needed to investigate this mechanism at the level of the airways, skin, and gut.

## 4. Conclusions

Given the rising prevalence of food allergies in urbanized and industrialized countries, a role for the damage of epithelial barrier as a pathogenetic mechanism has been hypothesized. In fact, exposure to harmful environmental factors associated with environment anthropization and industrialization (i.e., climate change as well as exposure to air pollutants, detergents, MPs and NPs, and food additives) can damage the epithelial barrier in the skin, gut, and airways with detrimental effects on the host immune response and on the local microbial equilibrium, resulting in chronic mucosal inflammation that perpetuates the dysfunction of the epithelial barrier. The increased epithelial permeability allows allergens to access the submucosae, leading to an imbalance between Th1 and Th2 inflammation with a predominant Th2 response, which is the key factor in food allergy development.

Further studies on the role of epithelial barrier dysfunction in food allergies are needed, particularly to deeply explore the specific contributions of epithelium damage at the level of the gut, skin, or airways. Moreover, the molecular pathways involved in epithelial dysfunction, as well as the role of external factors, need to be better understood. A more in-depth understanding of these aspects, in fact, can pose as the basis for the development of strategies to prevent or repair epithelial damage, with the final objective of reducing food allergy prevalence.
